# Does involvement in a cohort study improve health and affect health inequalities? A natural experiment

**DOI:** 10.1186/s12913-017-2016-7

**Published:** 2017-01-25

**Authors:** Annie Quick, Jan R. Böhnke, John Wright, Kate E. Pickett

**Affiliations:** 1 Department of Health Sciences, University of York, Heslington, York YO10 5DD UK; 2 Mental Health and Addiction Research Group (MHARG), Hull York Medical School and Department of Health Sciences, University of York, Heslington, York YO10 5DD UK; 3 Bradford Institute for Health Research, Temple Bank House, Bradford Royal Infirmary, Duckworth Lane, Bradford, BD9 6RJ UK

**Keywords:** Born in Bradford, Hawthorne effect, Health inequalities, Mere measurement

## Abstract

**Background:**

Evidence suggests that the process of taking part in health research can improve participants’ health, independent of any intended intervention. However, no research has yet explored whether these effects differ across socioeconomic groups. If the effect of mere participation in health research also has a social gradient this could increase health inequalities and bias research results. This study used the Born in Bradford family cohort (BIB) to explore whether simply taking part in BIB had improved participants’ health and, if so, whether this effect was mediated by socioeconomic status.

**Methods:**

Survey data on self-reported health behaviours were collected between 2007 and 2010 as part of BIB. These were augmented by clinical data on birth weight. Pregnant women on their second pregnancy, joining BIB for the first time formed the control group. Their health was compared to women on their second pregnancy who had both pregnancies within the study, who formed the exposed group. In order to limit the inherent bias in a non-randomised study, propensity score analysis was used, matching on age, ethnicity, education and date of questionnaire. The results were then compared according to mothers' education.

**Results:**

Of six outcomes tested, only alcohol consumption showed a statistically significant reduction with exposure to BIB (OR: 0.35, 95% CIs 0.13, 0.92). Although effect estimates were larger for women with higher education compared to lower education, these effects were not statistically significant.

**Conclusions:**

Despite one significant finding, these results overall are insufficient to conclude that simply taking part in BIB affected participants’ health. We recommend that socioeconomic status is considered in future studies testing effects of research participation, and that randomised studies with larger sample sizes are conducted.

## Background

Researchers have long been interested in whether the experience of being part of a research study can change the behaviour of participants. When these changes are due purely to the experience of being in a study — rather than as a result of any intervention — this is sometimes called a Hawthorne effect, measurement reactivity, or mere measurement effect [1–3]. Such an effect might occur as a result of filling in a questionnaire, being observed by a researcher, or taking part in an interview. This behaviour change might be the result of increasing participants’ awareness by asking about a health behaviour, or through prompting participants to reflect on their own choices.

Existing research suggests that being involved in a research study can produce statistically significant, though generally small effects in participants’ health behaviours. A recent meta-analysis examined the effect of asking questions on participants’ behaviour [4]. From 41 studies the authors found a small overall change in behaviour (Standardized Mean Difference = 0.09; 95% CI [0.04, 0.13]; k = 33) for those who had answered questionnaires or surveys compared to participants with no measurement, or those with other forms of measurement. However, studies did show publication bias, suggesting the effect size may be overestimated.

Another meta-analysis examined the mere measurement effect on a more homogenous sample of studies: randomised controlled trials of brief interventions for alcohol consumption [5]. The review identified eight nested trials carried out to assess the impact of measurement on alcohol use independent of the intervention. The meta-analysis did not find a statistically significant effect (*p* = 0.053), although the authors highlighted that the effect size was relatively large (those who had been measured at baseline drank 1.5 fewer units a week than those who had not), and statistical power was low. These randomised studies are particularly valuable as they avoid a number of biases included in observational studies, and more such studies would help to increase the power for meta-analyses and provide a fuller picture of the role of mere measurement.

In a narrative review, French and Sutton [2] found evidence of small, but significant effects from mere measurement. For example, one study was a cluster randomised controlled trial of physical activity promotion in which participants in the control and intervention groups were further randomised either to have measurements taken at baseline, eight weeks and six months, or just at six months. The measurement was a thirteen-page questionnaire, and height, weight and waist circumference were also measured at baseline. The number of people meeting the recommended exercise level was 50% higher in those who filled out the questionnaire three times, compared to those who filled it out once (OR: 1.5, 95% CI: 1.10, 2.06). No significant association was found, however, for the proportion of participants spending at least 150 min per week on physical activity [6].

Using patient information gathered via questionnaires is popular both in the areas of practice oriented research in psychotherapy [7] as well as in more general health contexts in order to collect patient reported outcomes [8]. In these settings repeated assessments during the course of care are obtained from the individual patients and generally these assessments, if fed back to clinicians, have been shown to improve treatment outcomes especially for patients that were on a trajectory of stagnation or even deterioration [9, 10]. Whether this effect is due to changes in practice or just the repeated assessments is a topic of current debate [11–13].

Many of the health behaviours examined in studies of mere measurement have a social gradient, whereby people who are more advantaged in income or education have better health behaviours than those lower down the social ladder. These include behaviours such as physical activity [6] or smoking [1]. However, there is almost no evidence of how, if at all, mere measurement differentially affects people of different socioeconomic status (SES). Given that many of the studies of mere measurement are nested within larger studies it is likely that data on SES were available, but this was rarely studied. One exception was found: a randomised controlled trial by van Sluijs et al., measuring the effect of surveying physical activity, found a significant change in behaviour due to measurement, but no significant change in the model when adjusting for confounders, among which were employment and education [6].

Very little research has been carried out exploring the causal processes behind mere measurement, beyond a general raising of awareness [2]. It is possible that mere measurement could prompt behaviour change through similar processes as health promotion activities. Clearly, the comparison is not exact; unlike mere measurement, health promotion generally involves the provision of information or explicit suggestions for behaviour change. However, it has been shown that information on its own is often ineffective at changing behaviour [14]. In the absence of direct studies on mere measurement, then, the way in which health promotion interacts with SES was examined.

One systematic review explored the effect of health promotion and education campaigns according to SES [15]. Studies examining the effects of accident prevention schemes, educational books for pregnant women and immunisation found that people with higher SES improved more, so that the overall improvement was at the expense of widening health inequalities. In the US, Pickett et al. found that the widely commended ‘Back to Sleep’ campaign to prevent Sudden Infant Death Syndrome (SIDS) not only increased race and SES inequalities in rates of SIDS, but the odds ratio for SIDS associated with lower SES actually increased during the campaign [16].

The history of smoking is one example of the inequitable effects of health promotion. Although there was no clear social gradient in smoking in the first half of the twentieth century, a social gradient emerged with the ‘Smoking Kills’ campaigns [17, 18]. Smoking prevalence changed very little in the lowest social class quintile between 1973 and 1998, whereas all other quintiles saw significant reductions [19]. An evaluation of England’s smoking cessation services in 2005 found a similar trend. Out of those smokers who took up smoking cessation services, disadvantaged groups had cessation rates of 8.7%, compared to a rate of 17.4% in the most advantaged groups [20].

If mere measurement and health promotion affect health behaviours through similar causal pathways then the negative effects of health promotion on equality raise the question of whether mere measurement, too, may increase health inequalities.

This study therefore aimed to address two sequential questions:

1. Does simply taking part in a research study improve participants’ health behaviour?
2. If so, is this effect mediated by socioeconomic status?

## Methods

### The Born in Bradford family cohort study

Born in Bradford (BIB) is a longitudinal multi-ethnic family cohort study aiming to examine the impact of environmental, psychological and genetic factors on maternal and child health and wellbeing [20]. Bradford is a city in the North of England with high levels of deprivation and ethnic diversity. Approximately half of the births in the city are to mothers of South Asian origin. Women were recruited while waiting for their glucose tolerance test, a routine procedure offered to all pregnant women registered at the Bradford Royal Infirmary, at 26–28 weeks gestation. For those consenting, a baseline questionnaire was completed via an interview with a study administrator^1^.

The baseline questionnaire for the mothers was transliterated into Urdu and Mirpuri using a standardized process, so that words and phrases corresponded with the original English version. As Mirpuri does not have a written form trained bilingual interviewers administered the transliterated questionnaires to Mirpuri speakers.

The full BIB cohort recruited 12,453 women during 13,776 pregnancies between 2007 and 2010 and the cohort is broadly characteristic of the city’s maternal population.

### Study design

During the course of the cohort study some women became pregnant more than once. These women were invited to include their additional babies in the cohort and fill out another baseline questionnaire. This feature of the cohort was used to assess the effect of BIB on mothers’ behaviour during pregnancy, as the health behaviours of mothers having their second baby in the cohort (who can be referred to as BIB P2s) could be compared with the health behaviours of mothers enrolled into BIB for the first time (who can be referred to as BIB P1s), who had not yet been exposed to the study. In our sample, health behaviours differed according to parity^2^. For example, using data from all BIB P1s, the odds of smoking during pregnancy decreased with each additional child, with an adjusted odds ratio of 0.86 (95% CIs 0.82, 0.91; *p* <0.001). We therefore restricted the study to include only women on their second pregnancies.

**Fig. 1:**
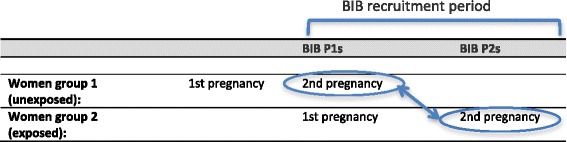
Study design

The study design is presented in Fig. 1. The control group is circled on the left hand side. These women had one pregnancy before BIB started, and a second child who was included in BIB. Their BIB P1 acts as our control. Their baseline questionnaire was filled out as they entered the study, so being part of the study should not have influenced their responses.

The exposed group is circled on the right hand side. These are women who had two children and both of them were included in the BIB study. They filled in the BIB questionnaire for each pregnancy. The data collected during their second pregnancy in the cohort acts as our exposed group because they have been exposed by filling out the BIB questionnaire during their first pregnancy (BIB P1). This study used only their responses from the questionnaire completed during their second BIB pregnancy. The data from their first BIB pregnancy was disregarded, so the control and exposed groups consist of different women.

### Outcomes and data collection

#### Health behaviours

The current official NHS advice is not to smoke, to consume only small quantities of caffeine during pregnancy and to take vitamin D supplements^3^. Women are also advised not to drink alcohol, although there is some leniency in the consumption of small quantities. Two measures for alcohol were therefore included to reflect differing levels of alcohol consumption. Five health behaviours were therefore analysed, all of which were dichotomous variables:

- **Smoking**. Women were coded yes if they reported smoking one or more cigarettes a day during pregnancy.
- **Drinking any alcohol**. Women were coded yes if they reported drinking any alcohol during the first three months of pregnancy.
- **5 or more units of alcohol once a month or more**. Women were coded yes if they reported drinking five or more units of alcohol 1–3 times a month or more during the first three months of pregnancy.
- **Caffeine consumption**. To reflect NHS advice that consuming small quantities of caffeine is acceptable, women were coded yes if they reported drinking more than one caffeinated drink per day, during the previous four weeks.
- **Vitamin D supplementation**. Women were coded yes if they reported consuming vitamin D, Pregnacare (Vitabiotics) or Sanatogen prenatal (Bayer), which both contain vitamin D, during the previous four weeks.

All five behavioural outcomes were collected as part of the BIB baseline questionnaire at 28 weeks gestation.

#### Clinical outcome

Given the potential biases in self-reporting, birth weight was also used as an objective measure to act as a check on the reliability of self-reported behaviours. Research suggests that the risk of low birth weight is increased by smoking [21], alcohol [22], and possibly caffeine [23]. There is also some evidence that vitamin D is preventative of low birth weight [24, 25].

Birth weight was a continuous variable and taken from the maternity IT system.

Missing data was negligible for all outcome variables (maximum 0.3%).

### Covariates

Estimates were adjusted for age in years, ethnicity (white British, Pakistani and other), mother’s education (<5 GCSE’s, 5 GCSEs, A Levels, higher than A Levels, other), and date of questionnaire.

The date of questionnaire was included because BIB P2s would occur, on average, later in the recruitment period than BIB P1s. A difference between BIB P1s and BIB P2s could therefore reflect changes in health behaviour in the population over time, rather than the effect of being part of BIB.

All covariates came from the BIB baseline questionnaire except mother’s age, which was obtained from the maternity record. These covariates were chosen a priori, on the basis that they would be associated with either health behaviour outcomes, or with the likelihood of a mother having two pregnancies within the BIB recruitment period.

### Statistical methods

Given the possibility of bias in the absence of randomisation, we used propensity score matching, which is increasingly recognised as a robust method for assessing exposure effects in a non-experimental study design [26].

The propensity score is the conditional probability of having been exposed, given a set of observed covariates [27]. In the current study, the propensity score was calculated based on a multiple logistic regression model predicting previous exposure to BIB. This way, each mother’s propensity score represented the probability that she was a BIB P2 (rather than a BIB P1) based on her age, ethnicity, education, and the date she filled out her questionnaire.

Nearest neighbour matching with common support was then used to create matched pairs. This involved firstly matching each BIB P1 in our control group with a BIB P2 with the closest propensity score. If there was more than one BIB P1 with an identical propensity score, one was chosen at random. Secondly, common support was applied, whereby BIB P2s whose propensity score was higher or lower than the range in propensity scores of the BIB P1s, and BIB P1s with a propensity score higher or lower than all BIB P2s were all excluded, ensuring that both groups had propensity scores within the same range. All the other BIB P1s were then dropped from the analysis to leave only the matched pairs.

A number of different matching techniques were tested, and nearest neighbour matching with common support was chosen because it created the closest matches. This process (called balancing) involved comparing the distribution of the propensity scores within the exposed and control groups, and also comparing the mean of each covariate in both groups. Ideally, the percentage difference in the means of the two groups (the percentage bias) should be less than 5%. In this case, none of the matching techniques were quite able to achieve this (see Appendix 1, Table 2 for the selected approach). The matching approach was chosen before doing the final logistic regression, i.e. blind to the effect of the matching method on the outcome.

Finally, a simple logistic regression was carried out within the matched cases only between the six behavioural outcome variables and exposure to BIB to obtain the relevant odds ratios testing our hypotheses. A simple linear regression was carried out for birth weight.

### Testing the differential effect of mere measurement by socioeconomic status

Mother’s education was chosen as the best available indicator of socioeconomic status. Education was dichotomised into those educated up to GCSE level, and those with higher levels of education. Separate logistic regressions were then carried out stratified by mother’s education, in order to compare results. Logistic regression was carried out using pairs matched on propensity score. Only outcomes showing the largest effects in the previous propensity score analysis were tested — any alcohol consumption and birth weight.

Significance levels for all analyses were two-sided and set at 5%. Stata 12 was used, with additional user-written programmes psmatch2 [28] and pscore [29] for propensity score matching.

## Results

### Summary statistics

Table 1 shows summary statistics of our control and exposed groups. BIB P1s, who had not yet participated in the study, were very slightly older (Z = 3.97, *p* < 0.001, d = .20) and filled out their questionnaire earlier (Z = −12.29, *p* < 0.001, d = .75) than BIB P2s.

**Table 1:** Baseline comparison: Covariates by exposure to BIB

	BIB P1s (unexposed) *N* = 1,411	BIB P2s (exposed) *N* = 225
Age in years		
Mean (SD)	29.60 (4.63)	28.28 (4.64)
Ethnicity (%)		
White British	32.24	28.89
Pakistani	54.69	59.11
Other	13.07	12.00
Education (%)		
<5 GCSEs	24.11	24.44
5 GCSEs	33.93	36.00
A-levels	13.73	10.67
Higher than A-level	20.27	24.44
Other	7.97	4.44
Questionnaire date Mean (SD)	659.98 (384.71)	999.48 (237.66)
Any alcohol (%)		
Yes	53.82	33.33
No	46.18	66.67
Alcohol – 5 units or more (%)		
Yes	25.00	25.00
No	75.00	75.00
Smoking (%)		
Yes	15.13	12.50
No	84.87	87.50
Vitamin D (%)		
Yes	15.10	9.33
No	84.90	90.67
Caffeine (%)		
Yes	61.30	59.11
No	38.70	40.89
Birth weight (grams) Mean (SD)	3268.28 (534.16)	3315.04 (496.00)

### Propensity score matching

Logistic regressions and balancing for propensity score matching are shown in appendix 1. Nearest neighbour matching produced a sample size of 156 matched pairs (*n* = 312).

Table 2 shows the results of logistic regression between BIB P1s and BIB P2s, matched on propensity score. When restricting to women on their second pregnancy, the only significant finding was for any alcohol consumption where the odds of drinking was 65% less for women who had been exposed to BIB compared to those who had only just joined the cohort (odds ratio 0.35, 95% CIs: 0.13, 0.92). The effect estimates of smoking and drinking five or more units of alcohol once a month or more were both in the direction of an improvement in health behaviours, and the effect estimate for birth weight also showed an increase. Effect estimates for vitamin D consumption and caffeine consumption were both marginally in the direction of a deterioration in health behaviours, though the effect sizes were very small (close to zero).

**Table 2:** Odds ratios and coefficient for exposure to BIB, propensity score matched pairs

	Odds Ratio (95% CIs)	*P* value
Smoking	0.75 (0.41, 1.38)	0.36
Any alcohol	0.35 (0.13, 0.92)	0.03
5 or more units of alcohol once a month or more	0.24 (0.03, 2.20)	0.21
Vitamin D	0.83 (0.42, 1.66)	0.60
Caffeine	1.05 (0.67, 1.66)	0.82
	Coefficient (95% CIs)	*P* value
Birth weight in grams	41.95 (−67.53, 151.44)	0.45

### Testing the differential effect of mere measurement by socioeconomic status

The previous section addressed the first research question: Has being part of BIB influenced participants’ health behaviour? This provided the basis for looking at the second question: Is the effect of participation in BIB on health moderated by socioeconomic status?

As many outcomes showed no significant change with exposure to BIB, just two outcomes were taken forward to test for mediation by socioeconomic status: any alcohol consumption (as it had shown a statistically significant difference) and birth weight, as an objectively measured variable.

Table 3 presents the result of two bivariate regressions each stratified by education, using pairs matched on propensity score. Although both outcomes showed a larger effect estimate for those with higher education than for those with lower education, neither was statistically significant.

**Table 3:** Propensity score matching: Odds ratio and coefficients for smoking, any alcohol consumption and birth weight by exposure to BIB, stratified by education, matched pairs

	Odds ratio by exposure to BIB (95% CIs)	
	5 GCSEs or fewer	A-levels or higher
Any alcohol	0.43 (0.12, 1.47)	0.26 [(0.05, 1.28)
	Coefficient by exposure to BIB (95% CIs)	
Birth weight	−39.25 (−186.04, 107.55)	161.22 (−69.14, 391.58)

## Discussion

This study did not provide conclusive evidence that a mere measurement effect consistently occurred in the BIB cohort study, or that it was moderated by socioeconomic status. Although some significant findings did emerge suggesting that further exploration of this topic is merited, the majority of tests showed no statistically significant effect.

1. **Has being part of Born in Bradford improved participants’ health behaviour?**
  Exposure to BIB was associated with a statistically significant improvement in health behaviours in only one of six regressions carried out — a significant reduction in the number of women reporting any alcohol consumption.
2. **If so, has this effect been moderated by socioeconomic position?**
  When cmstratifying by education, no significant effects were found either for any alcohol consumption, or for birth weight. However, effect estimates were larger for women of higher education, compared to women of lower education.

### Limitations

#### Non-randomisation

As a non-randomised natural experiment, this study design is vulnerable to confounding based on non-comparability of the control and exposed groups. Another approach to seeing if taking part in BIB affected health behaviour is a before and after study, comparing first and second BIB pregnancies for the same women. Because the same women are in the control and exposed groups, many potential cofounders are avoided. Such a before and after design would not be a good way to demonstrate whether BIB had made a difference, as there would be no way to distinguish behavioural changes that would occur anyway with increased numbers of children from changes due to participation in BIB. Nevertheless, if BIB had improved behaviour it should show up with this design. This analysis was therefore carried out as a sensitivity test and is shown in Appendix 2.

No significant difference was found in any health behaviour between women’s first and second pregnancy in BIB, although the effect sizes for both measures of alcohol use (OR 0.92 for any alcohol and 0.81 for 5 or more units of alcohol) indicate a non-significant reduction in alcohol consumption. A statistically significant difference in birth weight was identified, however. On their second BIB pregnancy, women had babies that were an average of 137.29 g larger than their first child within the BIB cohort, and this difference was statistically significant (95% CIs 35.52, 239.07). This finding does not rule out the possibility that there could be some modest improvement as a result of exposure to BIB, which does not reach significance in individual behaviours, but does amount to an overall improvement in mother’s health and therefore birth weight. However, the fact that these effect sizes are not large or significant, whereas our primary study design did show significance on one of the behavioural outcomes does suggest that those women who had two pregnancies within the cohort period may differ in unknown variables which are not possible to account for in propensity score matching.

#### The trade-off between robustness and power

Rather than adjusting for differences between the two groups, propensity score matching comes closer to imitating a randomised controlled trial by creating two groups that are as closely matched as possible, thereby only comparing observations that are similar enough to be comparable, based on known covariates [26, 30, 31]. This approach may also limit residual confounding; by only including in the analysis controls that have a similar propensity score, bias caused by any unknown variable that is a confounder *and* associated with one of the known covariates should be reduced.

However, the use of propensity score matching, as well as the restriction of the sample to second pregnancies involved a substantial loss of sample size and power.

There are some clues that a larger sample size may have been needed. Although only the regression testing for any alcohol consumption produced a significant finding, the estimated effect of exposure for BIB on smoking, consuming five or more units of alcohol once a month or more, and birth weight showed an improvement. The effect estimates for Vitamin D consumption and caffeine were in the direction of a deterioration, though both were close to zero.

When testing for moderation by socioeconomic status, significance was lost on any alcohol consumption for more and less educated women, but the effect estimates for both any alcohol and birth weight indicated a larger improvement in health behaviours for more educated, compared to less educated women.

Sample size is particularly important for studies of mere measurement where effect sizes are likely to be small [4], and for exploring moderation by socioeconomic status, where a still larger sample size may be required to assess more subtle distributional effects.

#### Outcome measures and fertility

Both alcohol consumption [32] and smoking [33] are known to reduce fertility. This creates a potential bias if it results in women who drink and smoke with their first child being less likely to have another child within the four year BIB recruitment period. If this were the case, BIB P2s would have improved health behaviour not because of the effect of BIB, but because of selection bias between the exposed and unexposed groups, leading to a possible type 1 error. However, as identified above, selection bias cannot fully account for those significant findings which were identified as the differences at baseline were smaller than those found after the BIB P2s had participated.

#### Recruitment of lower socioeconomic groups

Any studies in which participation is conditional on agreeing to take part in a survey are open to participation bias. In particular, studies may struggle to recruit participants of lower socioeconomic status. Analysis comparing the BIB cohort with all other births at the maternity department in Bradford Teaching Hospital found that the cohort did have marginally lower representation from mothers living in more deprived areas [34] [Table 1]. In the case that there was a difference in take-up between those of lower and higher education, the exclusion of those with very low socio-economic status could lead to a type 2 error.

### Further research

As we suggest above, there are theoretical reasons to expect that a mere measurement effect may be moderated by socioeconomic status. If this were the case, it would need to be taken into account in any long-term studies of health, and would also pose important ethical challenges for research studies, particularly those such as BIB that aim to decrease health inequalities. Although not conclusive, our findings suggest that this issue merits further study.

If such a question were to be incorporated into the design of a new research project, it could be designed as a nested RCT within an existing study, as described above in McCambridge and Kypri [5]. Such a design could overcome the challenges of selection bias found in this natural experiment and could easily incorporate an analysis of moderation with socioeconomic status. Ideally, such an analysis could incorporate more sensitive measures of socioeconomic status, including for example income, and explore how these different measures affected outcomes [35]. Crucially, the study should be powered not only to detect the small effect sizes expected from mere measurement, but also to detect differences between socioeconomic groups.

## Conclusion

Although there are many studies showing small effects from mere measurement, research has so far failed to explore whether these effects are moderated by socioeconomic status. Overall, this study did not find sufficient evidence to conclude that mere measurement effect had occurred in the BIB cohort study, or that it had been moderated by socioeconomic status. However, one out of six of our analyses showed significant effect, and effect estimates suggested that participation in BIB may be associated with larger positive health effects for women with higher education. Research using designs with more comparable control groups and a larger sample size and are needed in order to explore potential moderation with socioeconomic status.

## Figures and Tables

**Fig. 2:**
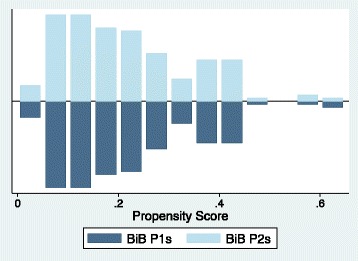
Distribution of propensity scores by matched BIB P1s and BIB P2s

## Appendix 1

### Balancing for propensity score matching

Table 4 presents the results of the logistic regression carried out in order to obtain the propensity scores. The outcome variable was exposure to BIB, and the odds ratios indicate the odds of being a BIB P2 according to each covariate.

**Table 4:** Propensity score matching: Odds ratios for exposure to BIB

	Adjusted Odds Ratios (95% CIs)^a^	Adjusted *p* value^a^
Age (per year increase)	1.01 (0.96, 1.07)	0.74
Ethnicity		
White British	Reference	
Pakistani	0.96 (0.58, 1.58)	0.52
Other	1.29 (0.60, 2.78)	0.60
Education		
<5 GCSEs	Reference	
5 GCSEs	0.72 (0.39, 1.33)	0.29
A-levels	0.81 (0.36, 1.84)	0.61
Higher than A-level	0.63 (0.32, 1.25)	0.19
Other	0.36 (0.13, 1.00)	0.050
Questionnaire date (per day increase)	1.00 (1.00, 1.00)	0.92

^a^Adjusted for age, ethnicity, education and questionnaire date

There were 225 BIB P2s on their second pregnancy. Only 156 of these BIB P2s were found appropriate matches due to only matching BIB P1s that were within the range of BIB P2s. Table 5 shows that after matching, the percentage bias was within 5% for all covariates except education which maintained a difference of -12.1%. Fig. 2 shows that the distribution of the propensity scores between BIB P1s and BIB P2s was adequately similar

**Table 5:** Comparison of means of covariates between BIB P1s and BIB P2s, before and after matching on propensity score

		Mean		% of bias
		BIB P2 (exposed)	BIB P1 (unexposed)	
Age	Unmatched	28.28	29.61	−28.7
	Matched		28.48	−4.4
Ethnicity	Unmatched	1.83	1.80	4.2
	Matched		1.81	3.5
Education	Unmatched	2.48	2.54	−4.7
	Matched		2.64	−12.1
Questionnaire date	Unmatched	999.48	661.77	105.7
	Matched		1012.6	−4.1

## Appendix 2

### Before and after sensitivity test

Table 6 presents regression analyses between women who participated in BIB twice in a before and after test. Their first pregnancy in the cohort acted as a control (BIB P1) while their second pregnancy acted as the intervention. Note that only women who had two pregnancies in the BIB cohort study were included in the control group.

**Table 6:** Before and after study of same women’s BIB P1 and BIB P2

Outcome variables:	Odds ratios for exposure to BiB	Adjusted *p* value
Smoking	0.98 (0.56, 1.71)	0.95
Any alcohol	0.92 (0.54, 1.57)	0.75
5 or more units of alcohol	0.81 (0.35, 1.88)	0.62
Vitamin D	0.71 (0.46, 1.11)	0.14
Caffeine	1.22 (0.86, 1.73)	0.26
Outcome variables	Coefficient for exposure to BIB	Adjusted *p* value
Birth weight	137.29 (35.52, 239.07)	0.008

## Abbreviations

BIB: Born in Bradford cohort study
NHS: National health service
OR: Odds ratio
SES: Socio-economic status
SIDS: Sudden infant death syndrome

## Footnotes

1 The baseline questionnaire can be found at http://www.borninbradford.nhs.uk/uploads/documents/questionaires/Born_in_Bradford_Mothers_Baseline_Questionnaire.pdf.

2 Strictly the difference between BIB P1s and BIB P2s is a difference if gravidity rather than parity as BIB P2s have not yet had their second live birth within BIB. For simplicity however, the term parity is used throughout.

3 Current NHS advice can be found at: http://www.nhs.uk/conditions/pregnancy-and-baby/pages/pregnancy-and-baby-care.aspx#close.
